# Solid Pseudopapillary Neoplasm of the Pancreas Misdiagnosed as Ovarian Torsion in the Second Trimester of Pregnancy

**DOI:** 10.7759/cureus.97306

**Published:** 2025-11-20

**Authors:** Sultan F Alanesi, Hassan Bahre, Shani Rahim, Fayes Abdullah, Marwan M Rasheed

**Affiliations:** 1 General Surgery, Khorfakkan Hospital, Sharjah, ARE; 2 Obstetrics and Gynecology, Khorfakkan Hospital, Sharjah, ARE

**Keywords:** multidisciplinary team, pancreatic tumors, pregnancy, solid pseudopapillary neoplasm, solid pseudopapillary neoplasm of the pancreas, tumor enucleation

## Abstract

Solid pseudopapillary neoplasm (SPN) of the pancreas is a low-grade malignant tumor affecting young women. Presentation during pregnancy is extremely rare and poses unique diagnostic and management challenges. We present a case of a 23-year-old primigravida at 25 weeks of gestation with recurrent epigastric pain and vomiting. Initial imaging suggested an adnexal mass, raising concerns about ovarian torsion. However, further assessment with MRI revealed a large heterogeneous mass anterior to the left kidney and near the pancreas. Diagnostic laparotomy confirmed a 15 cm mesenteric mass that was completely resected. Histopathological and immunohistochemical evaluations confirmed that the SPN originated in the pancreas. The patient recovered uneventfully and gave birth to a healthy, full-term infant. This case highlights the importance of SPN in the differential diagnosis of abdominal masses during pregnancy and illustrates the safety of second-trimester surgical management.

## Introduction

Solid pseudopapillary neoplasms (SPNs) are rare pancreatic tumors characterized by low malignant potential and a favorable prognosis. They predominantly affect young women but can also occur in men, though much less commonly. SPNs, often referred to as Hamoudi or Frantz tumors, are uncommon epithelial tumors of the exocrine pancreas with low malignant potential that occur mostly in younger women in the third and fourth decades of life [1]. They represent only 1-2% of all tumors of the pancreatic exocrine system [2]. The overexpression of progesterone receptors, found in 80%-100% of SPN cases, seems to contribute to the proliferation of tumor cells. This may account for the higher incidence of SPN in young females and the augmented tumor growth observed when serum progesterone levels are elevated, such as during pregnancy [2]. SPNs are generally asymptomatic and are found incidentally, with a large mass in the abdomen [1,3]. The clinical presentation is frequently nonspecific, with anemia, pain, or abdominal discomfort. Symptoms may be wrongly attributed to alterations during pregnancy in pregnant women, leading to delayed diagnosis [2].

Ultrasonography is usually the first test used in symptomatic patients and is also used for screening. Ultrasonographically, an SPN presents as a well-circumscribed hypoechoic cystic mass with sparse internal flow. Contrast-enhanced ultrasonography shows increased capsular borders with a nonenhanced center. In addition, iso/hypo-enhancement is observed in the early and delayed parenchymal perfusion phases [4,5]. Computed tomography (CT) is the most sensitive and commonly used scan for the diagnosis of pancreatic tumors [6].

Although surgical removal is the definitive management of SPNs and is typically curative, the timing and method must be thoroughly planned during pregnancy. The second trimester is regarded as the safest period for non-obstetric surgery because it poses a lower risk to the fetus [7]. This report describes a rare case of a large SPN that was initially misdiagnosed as ovarian torsion in a pregnant woman in her second trimester, emphasizing the importance of a wide differential diagnosis and multidisciplinary treatment.

## Case presentation

**Figure 1:**
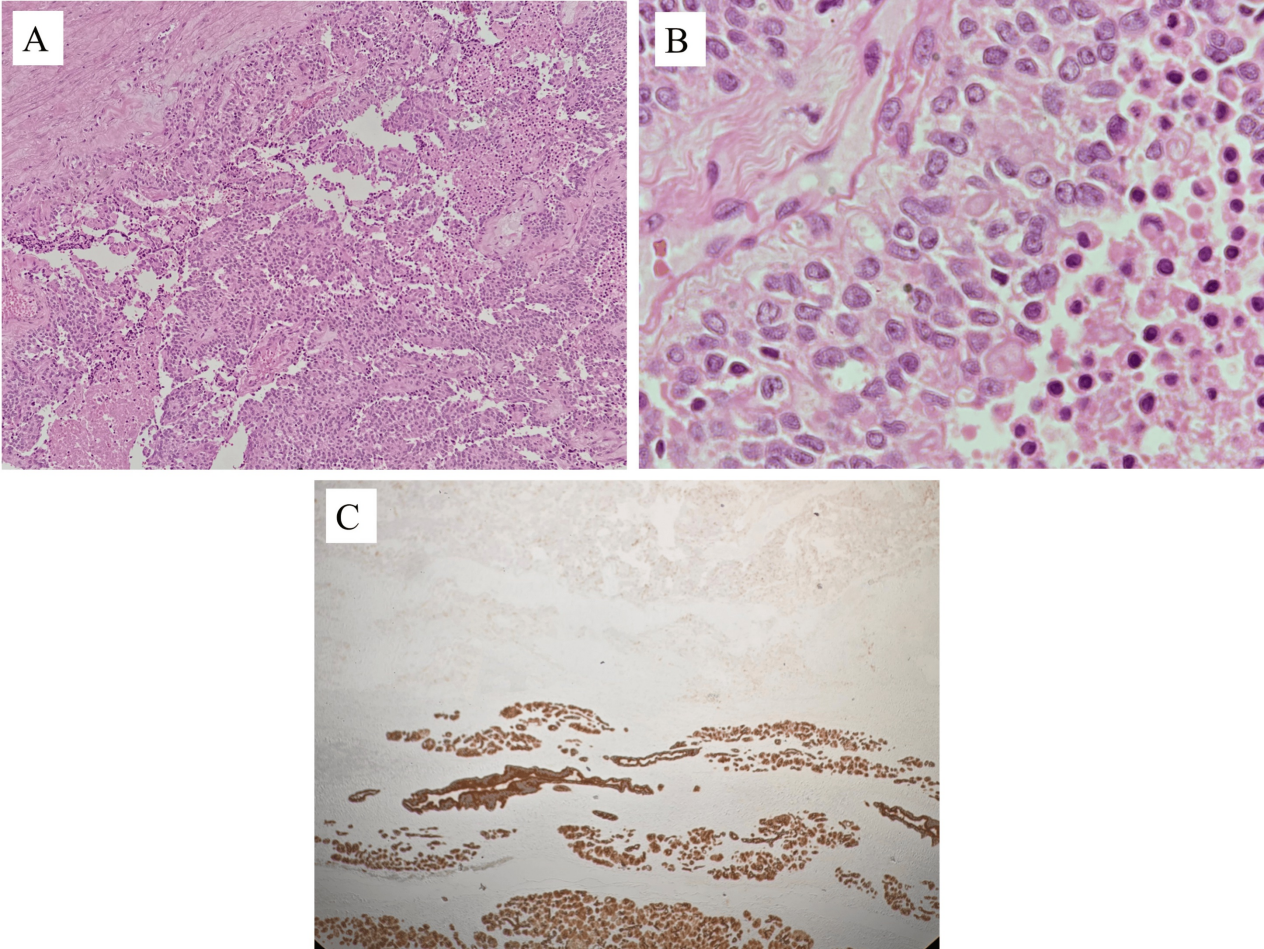
Histopathological images. A: Low-power view of the tumor shows solid and pseudopapillary growth with necrosis. B: High power view of the tumor cell lining. Tumor cells have a moderate amount of eosinophilic cytoplasm with relatively uniform nuclei with finely textured chromatin and inconspicuous nucleoli. C: Immunohistochemistry marker pan-CK (Ck Ae1/Ae3) was negative in the tumor and positive in the adjacent normal glands.

This case involves a 23-year-old primigravida patient at 25 weeks of gestation who presented with recurrent epigastric pain, nausea, and vomiting. The clinical and radiological investigations revealed a large mass in the epigastric region, which was initially suspected to be of ovarian origin (such as ovarian torsion). However, the definitive diagnosis was made following surgery and histopathological analysis, revealing an SPN of the pancreas (Figure 1).

Clinical presentation

The patient's symptoms progressively increased over the past week. The pain was described as sharp and severe, with a visual analog scale (VAS) score of 8/10. Initially intermittent, the pain became more persistent and was not relieved by routine analgesics. There was no history of trauma, urinary symptoms, or altered bowel habits. She had no record of undergoing abdominal surgery, experiencing any trauma, or having any related complaints.

Physical examination indicated that the patient was hemodynamically stable. Abdominal assessment showed slight tenderness in the epigastric region, with no signs of rebound tenderness or guarding. No peritonitis was observed. Obstetric assessment verified a live pregnancy within the uterus at the specified gestational age.

Laboratory investigations revealed a normal white blood cell count (reference range: 6-16 × 109/L) and mild anemia with a hemoglobin level of 10.2 g/dL (reference range: 13-17 g/dL). Her serum lipase level was 52 U/L, which was within the reference range of 16-77 U/L, suggesting that acute pancreatitis is unlikely.

Imaging studies

**Figure 2:**
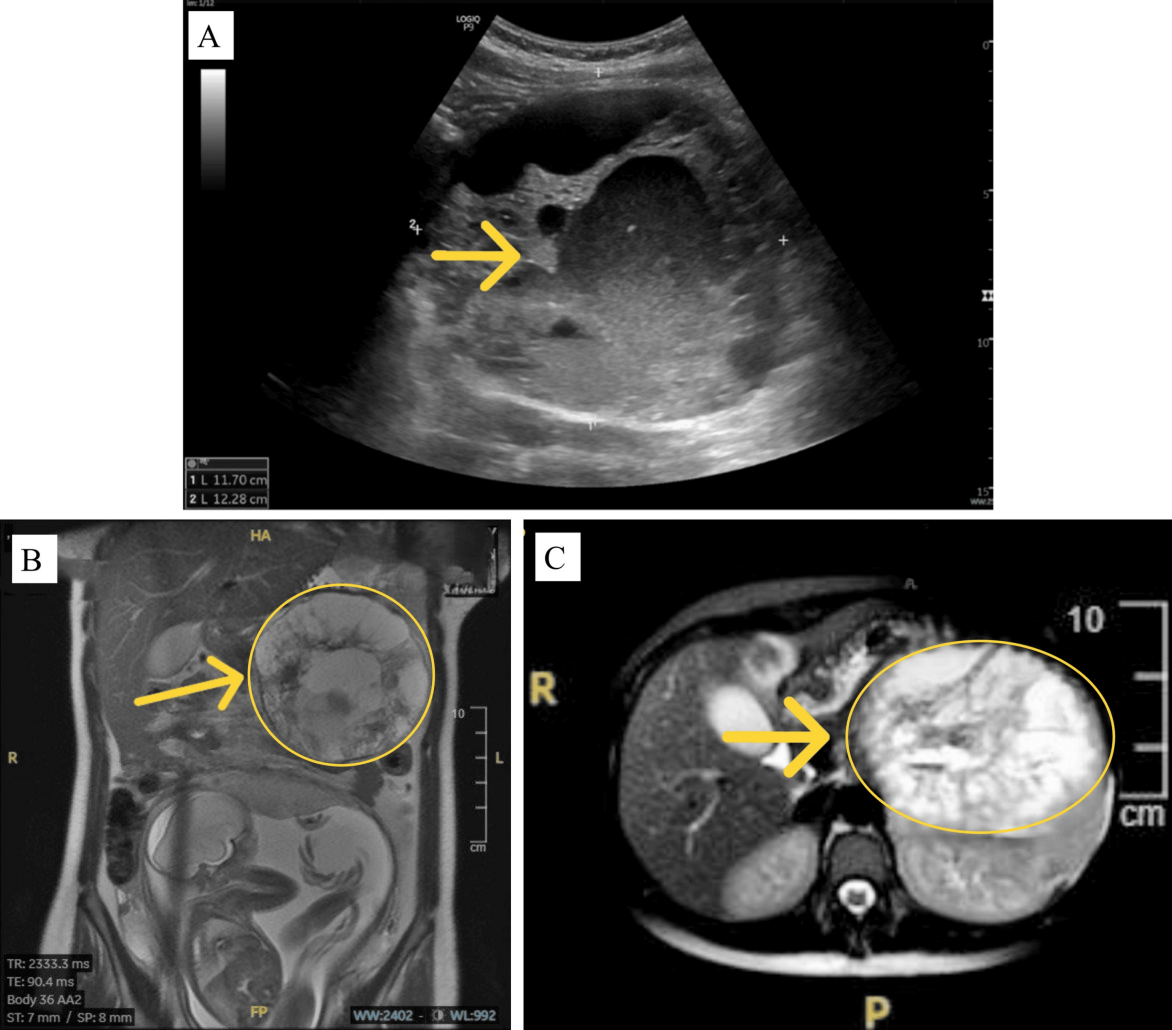
Ultrasound and MRI images of the abdomen before surgery. A: Abdominal ultrasound showing a large heterogeneous epigastric mass with cystic components (yellow arrow). B: Coronal T2-weighted MRI of the abdomen showing a large heterogeneous mass (yellow arrow) located anterior to the left kidney. The mass demonstrates mixed solid and cystic components, internal fluid levels, and peripheral hyperintense areas consistent with hemorrhagic or necrotic changes. It is displacing adjacent structures, including the pancreas and stomach. These imaging features were later confirmed to represent a solid pseudopapillary neoplasm (SPN) of pancreatic origin. C: Axial abdominal MRI scan demonstrating a large mixed solid-cystic tumor circled in yellow, displacing adjacent structures.

Abdominal ultrasound showed a large, well-defined, ovoid epigastric mass measuring approximately 12.9 × 11.4 cm. The lesion exhibited a heterogeneous echotexture with varying internal echogenicity and cystic spaces. The lesion was surrounded by a thick hyperechoic border, and multiple anechoic spaces within the mass indicated fluid accumulation. The mass was located anteriorly, and nearby structures were displaced. The pancreas could not be clearly visualized on ultrasound, as it was partially obscured by overlying midline structures and the gravid uterus. The yellow arrow in Figure 2A indicates the central cystic part of the lesion, which initially led to misidentification as a possible ovarian or adnexal mass. Although the mass was primarily epigastric, a possible ovarian or adnexal origin was considered in the differential diagnosis. This consideration was based on the effects of uterine enlargement displacing abdominal structures, the nonspecific nature of symptoms, and the unusual location of adnexal masses in pregnant women. Further imaging with MRI was recommended to clarify the origin and characteristics of the mass.

Subsequent MRI scans provided greater accuracy. The coronal T2-weighted MRI scan of the abdomen identified a sizable, well-capsulated heterogeneous mass anterior to the left kidney. The lesion was approximately 12 cm in the greatest dimension and had a mixed signal intensity with both solid and cystic elements. Internal fluid-fluid levels were noted, indicating hemorrhagic or necrotic changes in the mass. Areas of hyperintensity were noted peripherally, and the lesion was observed displacing neighboring structures, including the pancreas and stomach. The yellow arrow, as shown in Figure 2B, indicates the epicenter of the mass, which was one of the factors making the diagnosis difficult because the lesion simulated the appearance of an ovarian or gastrointestinal stromal tumor (GIST).

An axial MRI of the abdomen revealed an enlarged, well-circumscribed, heterogeneous mass circled in yellow in Figure 2C, anterior to the left kidney and next to the distal pancreas. The mass showed a mixed signal intensity with prominent solid and fluid-filled areas. Hyperintensity within the lesion indicated hemorrhagic or necrotic changes, and internal fluid-fluid levels were noted. The mass displaced the overlying structures, including the stomach and pancreas, without causing invasion.

Surgical intervention and diagnosis

Due to the size, unknown etiology, and symptomatic presentation of the mass, the patient was brought to the operating theater for exploratory laparotomy. The surgical team found a large mass in the mesentery, approximately 15 × 15 cm in size. It was located close to the medial colic artery on the left side of the mesocolon. Notably, the uterus, ovaries, and liver were all normal on gross inspection, and there were no signs of cancer dissemination within the cavity of the abdomen or any other abnormalities.

The surgical and obstetric team resected the entire mass and sent it for extensive pathological analysis. Microscopically, the tumor revealed the characteristic patterns of an SPN of pancreatic origin. These included pseudopapillary architectural patterns, cystic breakdown areas, monotonous neoplastic cells with eosinophilic cytoplasm, and hemorrhagic areas with foamy macrophages.

Additional immunohistochemical examination demonstrated that the tumor cells were positive for CD10, vimentin, progesterone receptor (PR), and neuron-specific enolase (NSE), and the low Ki-67 index indicated minimal proliferative activity. Notably, neuroendocrine and ovarian tumor markers were absent, proving that this neoplasm was of pancreatic origin, despite its aberrant site (Figure 1).

Outcome and follow-up

**Figure 3:**
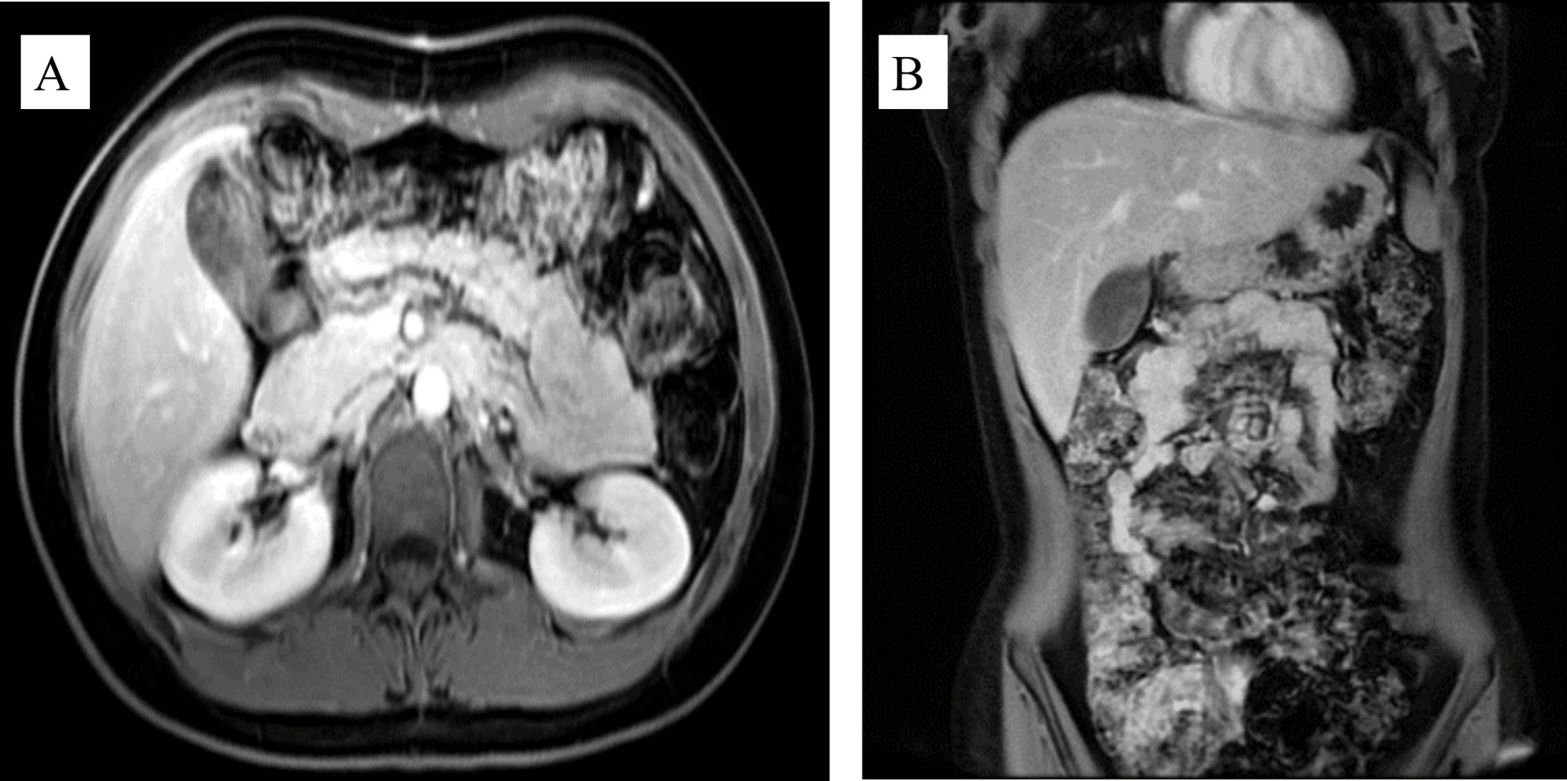
MRI of the abdomen after surgery. A: Axial T1-weighted post-contrast MRI image of the abdomen. Follow-up post-surgical excision of solid pseudopapillary neoplasm of the pancreas showing postsurgical changes in the pancreatic bed with no evidence of residual or recurrent mass. Surrounding abdominal structures appear unremarkable in this slice. B: Coronal T2-weighted MRI image of the abdomen: Follow-up post-surgical excision of solid pseudopapillary neoplasm of the pancreas, demonstrating postsurgical changes in the pancreatic bed with no evidence of residual or recurrent mass within the surgical site.

Following surgery, the patient recovered without complications and was discharged in a stable state on postoperative day five. Her pregnancy progressed smoothly without any complications, and she later delivered a healthy female infant at term via cesarean section. Follow-up assessments were conducted at one week, six months, and 12 months after discharge. Follow-up imaging using CT and MRI showed no residual tumor or evidence of recurrence. At her most recent follow-up, mother and infant were doing well (Figure 3).

## Discussion

Pancreatic SPN is an uncommon tumor, representing approximately 1-2% of all exocrine pancreatic tumors, and predominantly affects younger women in their 20s and 30s [8,9]. The present case demonstrates the diagnostic challenge of SPN during pregnancy. The initial ultrasound findings of a large, heterogeneous, cystic mass in the epigastric area led to its misinterpretation as an ovarian or adnexal mass, as reported by Zamboni et al. and Rajtar et al., with SPNs simulating gynecological neoplasms because of their similar imaging features [10,11]. Ultrasonography findings, featuring a notable central cystic area and a hyperechoic rim, added complexity to the diagnostic process.

In the present study, MRI scans demonstrated a heterogeneously encapsulated 12 cm mass with mixed solid and cystic components and internal fluid-fluid levels consistent with hemorrhage or necrosis. These characteristics are in line with earlier descriptions by Kovac et al. and Khristenko et al. [6,12], who observed that MRI is better at detecting the characteristic cystic degeneration and hemorrhagic zones of SPN. However, the atypical location of the lesion and its resemblance to GISTs or ovarian tumors complicated the preoperative diagnosis, similar to that noted in earlier studies [1,13].

During pregnancy, surgical treatments such as tumor enucleation or distal pancreatectomy can be carried out with favorable outcomes for both mother and infant [1,3]. Exploratory laparotomy in the present case uncovered a large mass in the mesentery adjacent to the medial colic artery, whereas the pancreas and reproductive organs were not involved, consistent with findings in ectopic or atypical pancreatic SPNs [14]. Histopathological and immunohistochemical analyses confirmed the diagnosis, revealing distinctive pseudopapillary structures, cystic degeneration, and strong reactivity to CD10, vimentin, PR, and NSE, which are well-documented features of SPN [15-17]. The overexpression of PRs, seen in up to 100% of SPNs, and nuclear accumulation of β-catenin have been implicated in hormone-sensitive tumor growth, which may explain the accelerated enlargement occasionally observed during pregnancy.

The physiological and anatomical changes during pregnancy may mask or simulate SPN symptoms, posing a diagnostic challenge. However, increased awareness and improved imaging methods have enabled these tumors to be identified more readily in pregnant individuals. In a case report and literature review by Naik et al., multiple cases of SPN during pregnancy were summarized, showing that the median tumor size at diagnosis was approximately 8-10 cm, and the majority of surgeries were performed in the second trimester, which is generally considered the safest period for non-obstetric surgery [2]. These findings reinforce the view that surgical intervention in the second trimester offers a favorable balance between minimizing fetal risk and ensuring timely maternal treatment.

In the current case, the patient underwent surgical resection in the second trimester, with favorable maternal and fetal outcomes. This aligns with the observations of Huang et al. and MacDonald et al., who reported successful resections of large pancreatic SPNs in pregnant women followed by term deliveries [1,18]. Collectively, these data suggest that when feasible, surgical excision of SPNs during the second trimester can be performed safely with good prognoses for both mother and child, provided that multidisciplinary management is ensured. The present case is consistent with the report by Al-Umairi et al., who emphasized the role of imaging in evaluating SPNs in pregnancy, and differs from the case reported by Santos et al., where distant metastasis was present, highlighting the advantage of early detection and complete resection prior to malignant transformation [8,19].

Furthermore, this case emphasizes the importance of maintaining a wide differential diagnosis when assessing abdominal masses in pregnant patients. Because the clinical and imaging presentation of SPN overlaps with more common conditions such as ovarian torsion, GISTs, or adnexal cysts, clinicians should consider SPN in younger women presenting with recurrent or unexplained abdominal pain. In the present case, the lesion’s location, size, and cystic appearance initially led to a false diagnosis of an adnexal or ovarian mass, a misinterpretation that could have led to suboptimal management had it not been corrected.

The incorporation of multimodal imaging, beginning with ultrasound and followed by MRI, was crucial in this patient’s diagnosis. The combination of solid-cystic morphology, fluid-fluid levels, and hemorrhagic components was highly suggestive of SPN. Histopathological confirmation after resection provided the definitive diagnosis. This case highlights the importance of a multidisciplinary approach involving obstetricians, radiologists, surgeons, and pathologists. A high index of clinical suspicion, along with appropriate imaging and histological assessment, can prevent misdiagnosis and facilitate timely management of this rare but significant condition during pregnancy.

## Conclusions

The present case illustrates the diagnostic dilemma of SPN in pregnancy when its clinical and radiologic presentation mimics more benign gynecologic disorders such as ovarian torsion. The successful outcome of second-trimester surgical removal of a sizable SPN, followed by full-term delivery of a healthy baby, reinforces the safety and efficacy of timely, multidisciplinary treatment. It also shows the necessity of a comprehensive differential diagnosis and the use of advanced imaging and histopathology to confirm diagnoses and achieve optimal maternal-fetal outcomes. Given the rarity of SPNs during pregnancy, there is a need for further research to establish optimal strategies for diagnosis, imaging, and surgical management of pancreatic tumors in pregnant patients. Prospective studies or multicenter registries could provide valuable insights into maternal and fetal outcomes, timing of interventions, and long-term prognosis.

## References

[1] Huang TT, Zhu J, Zhou H, Zhao AM (2018). Solid pseudopapillary neoplasm of pancreas in pregnancy treated with tumor enucleation: case report and review of the literature. Niger J Clin Pract.

[2] Naik RK, Amudhan A, Ashokkumar A, Inbasekaran A, Thangasamy S, Sathyanesan J (2024). Solid pseudopapillary epithelial neoplasm of pancreas in pregnancy: a case report and review of literature. Ann Hepatobiliary Pancreat Surg.

[3] Feng JF, Chen W, Guo Y, Liu J (2011). Solid pseudopapillary tumor of the pancreas in a pregnant woman. Acta Gastroenterol Belg.

[4] Fan Z, Li Y, Yan K (2013). Application of contrast-enhanced ultrasound in the diagnosis of solid pancreatic lesions--a comparison of conventional ultrasound and contrast-enhanced CT. Eur J Radiol.

[5] Xu M, Xie XY, Liu GJ (2012). The application value of contrast-enhanced ultrasound in the differential diagnosis of pancreatic solid-cystic lesions. Eur J Radiol.

[6] Kovac JD, Djikic-Rom A, Bogdanovic A, Jankovic A, Grubor N, Djuricic G, Dugalic V (2023). The role of MRI in the diagnosis of solid pseudopapillary neoplasm of the pancreas and its mimickers: a case-based review with emphasis on differential diagnosis. Diagnostics (Basel).

[7] Okeagu CN, Anandi P, Gennuso S (2020). Clinical management of the pregnant patient undergoing non-obstetric surgery: review of guidelines. Best Pract Res Clin Anaesthesiol.

[8] Al-Umairi RS, Kamona A, Al-Busaidi F (2015). Solid pseudopapillary tumor in a pregnant woman: imaging findings and literature review. Oman Med J.

[9] Chhabra D, Daver RG (2017). Solid pseudopapillary epithelial neoplasm of pancreas in pregnancy: case report of a rare co-occurrence. J Med Sci Clin Res.

[10] Zamboni GA, Ambrosetti MC, Pecori S, Manfredi R, Capelli P (2014). Solid pseudopapillary neoplasms. Imaging and Pathology of Pancreatic Neoplasms.

[11] Rajtar KZ, Sznajder K, Milto KM (2016). Diagnostic imaging of a solid pseudopapillary tumour of the pancreas in a 20-year-old woman - a case study. Prz Gastroenterol.

[12] Khristenko E, Gaida MM, Tjaden C (2024). Imaging differentiation of solid pseudopapillary neoplasms and neuroendocrine neoplasms of the pancreas. Eur J Radiol Open.

[13] Xu YC, Fu DL, Yang F (2024). Unraveling the enigma: a comprehensive review of solid pseudopapillary tumor of the pancreas. World J Gastrointest Oncol.

[14] Gong XH, Xu JR, Qian LJ (2021). Atypical and uncommon CT and MR imaging presentations of pancreatic ductal adenocarcinoma. Abdom Radiol (NY).

[15] Klimstra DS, Wenig BM, Heffess CS (2000). Solid-pseudopapillary tumor of the pancreas: a typically cystic carcinoma of low malignant potential. Semin Diagn Pathol.

[16] Omiyale AO (2021). Solid pseudopapillary neoplasm of the pancreas. World J Hepatol.

[17] Lu X, Chen H, Zhang T (2024). Solid pseudopapillary neoplasm (SPN) of the pancreas: current understanding on its malignant potential and management. Discov Oncol.

[18] MacDonald F, Keough V, Huang WY, Molinari M (2014). Surgical therapy of a large pancreatic solid-pseudopapillary neoplasm during pregnancy. BMJ Case Rep.

[19] Santos D, Calhau A, Bacelar F, Vieira J (2020). Solid pseudopapillary neoplasm of pancreas with distant metastasis during pregnancy: a diagnostic and treatment challenge. BMJ Case Rep.

